# Low-density lipoprotein cholesterol lowering treatment: the current approach

**DOI:** 10.1186/s12944-020-01275-x

**Published:** 2020-05-06

**Authors:** Irina Crismaru, Anca Pantea Stoian, Ovidiu Gabriel Bratu, Mihnea-Alexandru Gaman, Ana Maria Alexandra Stanescu, Nicolae Bacalbasa, Camelia Cristina Diaconu

**Affiliations:** 1 Emergency Institute for Cardiovascular Diseases “C.C. Iliescu”, Bucharest, Romania; 2 Department of Diabetes, Nutrition and Metabolic Diseases, Faculty of General Medicine, “Carol Davila” University of Medicine and Pharmacy, Bucharest, Romania; 3 “Carol Davila” University of Medicine and Pharmacy, “Carol Davila” University Emergency Central Emergency Military Hospital, Academy of Romanian Scientists, Bucharest, Romania; 4 “Carol Davila” University of Medicine and Pharmacy, “I. Cantacuzino” Clinical Hospital, Bucharest, Romania; 5 Department of Internal Medicine, “Carol Davila” University of Medicine and Pharmacy, Clinical Emergency Hospital of Bucharest, 8 Eroii Sanitari Blvd, 050474 Bucharest, Romania

**Keywords:** Dyslipidemia, LDL-cholesterol, Atherosclerosis, Statins, PCSK9 inhibitors

## Abstract

In the last 50 years, several clinical and epidemiological studies during have shown that increased levels of low-density lipoprotein cholesterol (LDLc) are associated with the development and progression of atherosclerotic lesions. The discovery of β-Hydroxy β-methylglutaryl-CoA reductase inhibitors (statins), that possess LDLc-lowering effects, lead to a true revolution in the prevention and treatment of cardiovascular diseases. Statins remain the cornerstone of LDLc-lowering therapy. Lipid-lowering drugs, such as ezetimibe and bile acid sequestrants, are prescribed either in combination with statins or in monotherapy (in the setting of statin intolerance or contraindications to statins). Microsomal triglyceride transfer protein inhibitors and protein convertase subtilisin/kexin type 9 (PCSK9) inhibitors are other drug classes which have been investigated for their potential to decrease LDLc. PCSK9 have been approved for the treatment of hypercholesterolemia and for the secondary prevention of cardiovascular events. The present narrative review discusses the latest (2019) guidelines of the European Atherosclerosis Society/European Society of Cardiology for the management of dyslipidemia, focusing on LDLc-lowering drugs that are either already available on the market or under development. We also consider “whom, when and how” do we treat in terms of LDLc reduction in the daily clinical practice.

## Introduction

The association between dyslipidemia and cardiovascular atherosclerotic disease is well established. In the last 50 years, a number of clinical and epidemiological studies have shown that increased levels of LDL cholesterol (LDLc) and low levels of HDL cholesterol (HDLc) correlate with the development and progression of atherosclerotic lesions. The discovery of β-Hydroxy β-methylglutaryl-CoA (HMG CoA) reductase inhibitors (statins) truly revolutionised the prevention and treatment of cardiovascular diseases. In the years that followed the introduction of statins in clinical practice, the management of dyslipidemia was mostly based on these drugs. Recently, several drug classes with cholesterol-lowering effects have been tested and approved for the treatment of dyslipidemic patients in whom conventional therapy (statins, ezetimibe, and bile acid sequestrants) did not efficiently control lipid values. Such drugs include anti-pro-protein convertase subtilisin/kexin type 9, apolipoprotein(a) antisense oligonucleotide and microsomal triglyceride transfer protein inhibitors.

As clinicians, the main questions we ask ourselves when managing dyslipidemic patients are: “Whom do we treat?”, “When is the initiation of a pharmacological agent justified?” “When do we consider the treatment to be effective and when do we need to change our approach?” and “What is the optimal treatment and which drugs do we use?”

In this narrative review, we focused on “whom, when and how” do we treat in terms of LDLc reduction in the daily clinical practice. This approach will help physicians to efficiently reduce the cardiovascular risk of their patients via lipid profile improvement. Also, we present LDLc reduction strategies in some particular clinical settings, such as chronic kidney disease, autoimmune disorders and elderly patients, as well as a short description of the new emerging LDLc-lowering drugs that are in the pharmaceutical pipelines or in different stages of clinical trials.

### Whom do we treat?

The decision to start lipid-lowering treatment in a specific patient is based on the analysis of lipid fractions (the ones associated with a high cardiovascular risk) and its correlation with the presence of other cardiovascular risk factors, as well as the analysis and estimation of the total cardiovascular risk.

Strong evidence, derived from multiple studies, shows that the reduction of LDLc using statin treatment leads to a significant decrease in the cardiovascular risk, both in terms of primary prevention, as well as in the secondary prevention of cardiovascular events [1, 2]. Despite the fact that statins reduce the cardiovascular risk by 15 up to 37%, a substantial residual risk of 60–80% still remains [3]. This residual risk is due to an inadequate LDLc reduction, low levels of HDLc and high levels of triglycerides (TG) [4, 5].

The baseline lipid evaluation includes total cholesterol, HDLc, LDLc, TG, non-HDLc and the total cholesterol/HDLc ratio. The latest European guidelines for the management of dyslipidemia recommend that LDLc levels should be the main target of dyslipidemia treatment [6]. The secondary treatment targets are non-HDLc and apolipoprotein B (apoB), because these lipid fractions have not been extensively studied in randomized, controlled clinical trials. However, this hierarchy is disputed. Of particular interest to researchers is apoB, which seems to predict cardiovascular risk as well as LDLc or more accurately [7]. One meta-analysis showed the superiority of apoB over non-HDLc and LDLc and concluded that among these three lipid fractions, LDLc was the weakest predictor of cardiovascular risk [8]. Moreover, LDLc cannot be accurately used to estimate the concentration of LDL particles when the patient also suffers from hypertriglyceridemia, a disadvantage that can be avoided by dosing apoB.

The estimation of the total cardiovascular risk is based on the idea that the main atherogenic lipid component is cholesterol. This risk, however, seems to be correlated more with the number of atherogenic particles (each one containing an apolipoprotein B molecule) that penetrate the arterial wall, rather than the cholesterol concentration of these fractions [7]. Therefore, apoB measurement seems to be more accurate in estimating the atherogenic burden. Moreover, apoB seems to be more reliable in the assessment of residual risk and treatment efficiency in the patients who receive lipid-lowering drugs. Statins are the first-line treatment in the reduction of LDLc and apoB levels. Studies which have evaluated the LDLc and apoB response to statin therapy have shown a higher reduction in LDLc than apoB levels following prescription of statins [9]. The evaluation of treatment efficacy based solely on LDLc inevitably leads to the under-treatment of dyslipidemic patients, as the number of atherogenic particles is higher than the one predicted using only the levels of LDLc. This hypothesis is supported by the results of multiple studies, concluding that the level of LDLc during treatment is not a reliable predictor of the residual cardiovascular risk, unlike the level of apoB in patients undergoing treatment [10, 11]. These results favor the use of apoB as a therapy target, since reaching the target level of LDLc does not seem to suffice. Many patients with “controlled” LDLc have a persistently high concentration of atherogenic particles. In such cases, an additional benefit may arise from treatment adjustment, e.g. an increase in the statin dose or replacing it with a more potent statin.

Non-HDLc has also been suggested as a treatment target in dyslipidemia. It is used as an estimation of the total amount of atherogenic lipoproteins in the plasma, since it correlated with apoB levels [12]. This variable is easy to compute, by subtracting HDLc from the total cholesterol, thus avoiding the measurement of apoB which is a costly investigation on a large scale. However, this proposal is still debated [13].

Plasma levels of HDLc have a significant reverse correlation with the incidence of cardiovascular events. The main mechanism for cardiovascular protection is the ability of HDLc to promote cholesterol efflux from cells, and especially from macrophages. Cholesterol is then transported to the liver and excreted into the bile. Treatment strategies to raise HDLc levels seem a reasonable approach in decreasing cardiovascular risk and this is a major objective when new hypolipidemic agents are developed. However, this approach has never been objectively proven in large clinical studies. On the contrary, studies involving drugs that raise HDLc did not results in a reduction of the cardiovascular risk or of mortality, as proven by several meta-analyses [14]. However, the interest for HDLc did not disappear, but it shifted towards exploring the function of HDLc. The cholesterol efflux capacity refers to the ability of the patient’s HDLc to promote cholesterol efflux from macrophages, and this correlates with the prevalence and incidence of cardiovascular diseases independently of the HDLc level [15]. This theory could explain the failure of therapies that raise the level of HDLc but do not influence its function. The reconsideration of the initial hypothesis, from this perspective, of a dysfunctional HDLc, led to studies involving new pharmacological agents able to promote cholesterol efflux and which are currently in progress.

The role of TG as a cardiovascular risk marker was strongly debated, as TG are frequently assessed as part of the lipid profile and can be complexly altered. High levels of TG are usually associated with a low level of HDLc and high levels of small dense LDLc particles, but they seem to predict cardiovascular risk also independently [16]. The treatment of hypertriglyceridemia seems to be beneficial in patients with high cardiovascular risk and triglycerides > 200 mg/dL and low levels of HDLc (< 40 mg/dL) [6]. The currently employed and recommended treatments of hypertriglyceridemia have a number a limitations that prevent reaching the target goals set for these patients in a high number of instances. However, novel therapeutic molecules might address this issue. Modified PPARα agonists are currently studied and might emerge as pharmacological agents that not only improve the atherogenic lipoprotein profile, significantly lowering lipoproteins rich in TG, but also safely reduce the residual cardiovascular risk [17].

### When do we treat?

According to the current European guidelines’ recommendations, the evaluation of patients and the targets of treatment should be based on the most common European score of cardiovascular risk assessment – SCORE (Systematic Coronary Risk Evaluation). This model, first introduced in the European guidelines in 2003, was built retrospectively based on cohorts from the time frame 1967–1991. The predictive factors included were considered age, sex, smoking, systolic arterial pressure and total cholesterol, and the events analyzed (fatal cardiovascular events) were established based on the limited available data. The threshold for defining high risk remained the same as in the 2003 guidelines – 5% for 10-year risk of fatal cardiovascular disease [18]. This threshold was considered to be the equivalent of 20% for the 10-year risk of fatal and non-fatal coronary events, the threshold for high cardiovascular risk in the previous guidelines. However, no rationale was provided for choosing these thresholds [18]. Moreover, unlike the American cardiovascular risk assessment model, Pooled Cohort Equations (PCE), SCORE is a model for predicting mortality, but without taking into account morbidity. Since this score risk was designed to be used in the primary prevention of cardiovascular events, it seems logical to consider the onset of a first cardiovascular event, and not a fatal event, since secondary prevention has become more and more effective, increasing the survival of patients with cardiovascular diseases.

On the other hand, the American PCE is also an inaccurate instrument, since it is based on outdated cohorts. One of the analyses on population categories shows that PCE overestimates cardiovascular risk by an average of 10–20% on risk groups, especially in Afro-American patients. This means that more than 10 million patients, currently listed in the high-risk category, should be reassessed and relocated to the low-risk category [19].

In patients with very high cardiovascular risk (patients with established cardiovascular disease, with moderate or severe chronic kidney disease, diabetes with organ damage or SCORE > 10%), the LDLc goal is < 55 mg/dL or a reduction of at least 50% from baseline. This goal can be even lower (< 40 mg/dL) for patients with cardiovascular disease who experience a second vascular event within 2 years, while treated with the maximum tolerated dose of statins. The LDLc goal for patients with high cardiovascular risk (SCORE = 5–10%) is < 70 mg/dL (or a reduction of at least 50% from baseline), and for those with moderate risk (SCORE = 1–5%) less than 100 mg/dL [6].

For patients with acute coronary syndromes, no particular approach is recommended – these patients are considered at very high cardiovascular risk, and the European Society of Cardiology recommends a “target to treat” strategy. The goal is an LDLc below 70 mg/dL or a reduction of at least 50% from the baseline if LDLc is between 70 and 135 mg/dL. The treatment must be initiated in the first 1–4 days of hospitalization due to an acute coronary syndrome and the efficiency or the safety of treatment should be assessed 4 to 6 weeks after the event [6]. On the contrary, the American guidelines, as well as the Japanese ones, recommend a “fire and forget” approach, based on a maximum intensity statin treatment, irrespective of the LDLc levels.

The arguments that support the dramatic reduction in LDLc immediately after an acute coronary event are the proven reduction of subsequent cardiovascular events, the pleiotropic effects of statins and the stabilization of atheroma plaques. Combining classical and novel therapies might be an option for an individualized treatment in some patients. Pro-protein convertase subtilisin/kexin type 9 (PCSK9) inhibitors have been studied in patients with acute coronary syndromes only after 2–3 months of treatment with statins and never in the acute phase (first month) of an acute coronary syndrome. Studies suggest that the level of PCSK9 is high in the acute phase of a coronary event due to inflammatory purposes, suggesting that an early initiation of PCSK9 inhibitors might be justified [20].

### How much do we treat?

The “lower is better” principle has already been proven by multiple studies. A 2010 meta-analysis of statins versus control (21 studies) and high-intensity statin treatment versus low-intensity statin treatment (5 studies) showed a reduction of the general mortality by 10% for a 1 mmol/L reduction in LDLc, and a subsequent decrease in mortality from coronary disease and other cardiac causes [21].

The possibility to drastically reduce cholesterol levels led to the question: how safe is such a brutal reduction? At what level should we be concerned regarding the cholesterol-dependent metabolic functions, such as the synthesis of gonadal hormones and the adrenal function? And what about fat-soluble vitamins? Moreover, some studies have shown a higher rate of cataract or cataract interventions after statin treatment. Randomized studies with alirocumab did not confirm a significant difference between the treated group and the control group regarding musculoskeletal and neurological events (including peripheral neuropathy), neurocognitive events, renal and hepatic events. The incidence of cataract was indeed higher in patients with LDLc < 20 mg/dL. The follow-up interval of these patients was only 104 weeks and the long-term effects need to be further studied [22].

### What do we use? Newly approved treatments

Statins play the main role in the treatment of dyslipidemia, but the significant residual cardiovascular risk despite statin treatment has driven the continuous quest for new drugs. The development of PCSK9 inhibitors and their inclusion in the European guidelines of 2016 represented an improvement in the management of dyslipidemia.

PCSK9 is a protein secreted by the liver, which binds the LDLR-LDL complex from the hepatocyte and promotes the transfer of LDLR (low density lipoprotein receptor) to the lysosomes for degradation, instead of allowing it to be recycled to the surface of the cell. In this way, PCSK9 reduces the level of LDLR from the surface of the hepatocytes, limiting the uptake and the degradation of LDL. Different approaches for PCSK9 inhibition have been proposed – monoclonal antibodies, small interfering ARN, antisense oligonucleotides and mimetic peptides. Of these, only monoclonal antibodies have shown positive results in human studies.

The first human study about the efficiency of an anti-PCSK9 antibody, alirocumab, was published in 2012 and showed a reduction of LDLc of almost 60% in the highest dose [23]. Phase 3 studies proved that the reduction of LDLc is correlated with a decrease in the occurrence of myocardial infarction, stroke, hospitalizations due to unstable angina and myocardial revascularization [24]. Considering these significant LDLc and cardiovascular mortality reductions, the introduction of PCSK9 inhibitors on a large scale, for the primary and secondary prevention of cardiovascular diseases, seems rational. However, their use is limited: the European guidelines recommend PCSK9 inhibitors for patients with persistently high LDLc levels (despite treatment with the maximum tolerated dose of statins in combination with ezetimibe) – class I – or for patients with statin intolerance (class IIB), as well as in the setting of familial hypercholesterolemia with very high cardiovascular risk (class IC) [6]. Their underuse is related mainly to the high costs and the administration by subcutaneous injections [25].

Two anti-PCSK9 monoclonal antibodies have been currently approved – alirocumab and evolocumab - with similar results. The study of a third antibody, bococizumab, has been interrupted in 2016, after 10–15% of patients developed anti-bococizumab antibodies (to note that this monoclonal antibody was chimeric, unlike the other two, that were human), and the reduction of LDLc had a very high variability, even in patients who had not developed antibodies [26].

The European Society of Cardiology/ European Society for Atherosclerosis released in 2017 a practical guideline for the use of PCSK9 inhibitors in patients with atherosclerotic cardiovascular disease or familial hypercholesterolemia [25]. The goal was to define the category of patients with a very high risk of cardiovascular events and to define an LDLc threshold for the initiation of treatment with anti-PCSK9 antibodies. In patients with atherosclerotic cardiovascular disease, the consensus recommends treatment with anti-PCSK9 at LDLc values higher than 140 mg/dL, a value that needs a 50% reduction to reach the recommended target [27]. The presence of severity factors – rapidly progressive atherosclerotic disease – lowers this threshold to 100 mg/dL. In patients with familial hypercholesterolemia without cardiovascular disease, PCSK9 inhibitors can be considered if LDLc is higher than 200 mg/dL, or even lower, 175 mg/dL, if there are additional severity factors [27]. PCSK9 inhibitors may be considered in these patients after an initial treatment with the maximum tolerated dose of statins, with the evaluation of the LDLc response after at least 4 weeks of therapy. If the goal is not reached, ezetimibe may be introduced, with a second re-evaluation after 4 weeks [27]. Only if the goal has not been reached yet, anti-PCSK9 treatment can be initiated. The first assessment of LDLc can be made after 2 weeks.

Although patients with severe chronic kidney disease are considered to have a very high cardiovascular risk, the lack of data from clinical studies does not allow the recommendation of anti-PCSK9 medication in this category of patients.

### Promising new molecules

The PCSK9 path can also be explored to identify other therapeutical targets. PCSK9 is mainly produced by the liver, so pharmacological agents that interfere with the hepatic production of PCSK9 can offer an alternative to monoclonal antibodies. Of these, the most promising so far seems to be inclisiran. Inclisiran is an artificially synthesized molecule of small interfering RNA (siRNA), recently employed to reduce the level of PCSK9 [28]. SiRNA interferes with the expression of particular genes with complementary nucleotide sequences, leading to the degradation of mRNA post-transcription, preventing thus translation. So far, the molecule seems to significantly reduce the level of PCSK9 and LDLc. Their advantages, compared to anti-PCSK9 monoclonal antibodies, are the biannual administration (versus 12–26 injections/year for PCSK9 inhibitors) and their intracellular mechanism of action which directly involves the hepatocytes (unlike the plasmatic activity of PCSK9 inhibitors). The ongoing trial ORION11 will establish the benefits regarding cardiovascular risk, as well as the safety profile of this drug.

Bempedoic acid is a non-statin new molecule that decreases LDLc by inhibiting the ATP citrate lyase, a key enzyme in the cholesterol synthesis pathway [29]. Bempedoic acid is the first oral, once-daily, non-statin LDLc-lowering drug approved by the FDA in the United States for patients with heterozygous familial hypercholesterolemia or with established atherosclerotic cardiovascular disease, already treated with a maximally tolerated dose of statins and who need further LDLc lowering [30]. This new agent has been studied in four clinical trials involving more than 3600 patients. The results of one of the latest trials, CLEAR Wisdom, have been presented at the American College of Cardiology Congress in 2019, concluding that the addition of bempedoic acid in patients already treated with a maximally tolerated dose of statins led to a significant reduction of LDLc (15.1%) after 12 weeks of treatment. Moreover, total cholesterol, non-HDLc, apoB and C-reactive protein levels have been reduced [31]. Bempedoic acid is currently being studied in the CLEAR Outcomes trial whose results are expected to be released in 2022.

Gemcabene calcium is another oral molecule evaluated in experimental studies on rats for its lipid-lowering effects which are independent of PPARs. This molecule decreased LDLc, TG, and apolipoprotein C-III levels in male rats [32] and is currently studied in humans.

Patients who present a very low response to statin and PCSK9 inhibitors treatment (both of which need a high activity of LDLR as a mediator for their effects) are those affected by homozygous familial hypercholesterolemia caused by mutations of LDLR on both alleles. This particularity led to the development of two therapeutical approaches which do not require LDLR but reduce LDLc by decreasing the hepatic production of VLDL (very low-density lipoproteins).

Lomitapide is a molecule that inhibits the TG microsomal transfer protein necessary for the assembly and secretion of VLDL from the liver. In patients with familial hypercholesterolemia, lomitapide reduced the level of LDLc by 50% after 26 weeks of treatment. The HDLc level dropped after 6 weeks, but returned to the pre-treatment level at 78 weeks. The most important disadvantage is related to the mechanism of action – the reduction of VLDL secretion leads to the accumulation of lipids in the liver, reflected also in the elevation of liver enzymes [33]. The risk-benefit ratio is favorable in these patients and lomitapide was approved in Europe in 2013 as an orphan drug strictly for the treatment of familial hypercholesterolemia.

Another orphan drug for the treatment of familial hypercholesterolemia is mipomersen, a synthetic antisense oligonucleotide that binds to the apoB RNA, inhibiting the production of apoB [34]. Mipomersen reduces the level of LDLc by approximately 25% in patients with familial hypercholesterolemia and was approved by the FDA in 2013. Unfortunately, the hepatic toxicity was considered too important by the EMA. This, along with the high number of cardiovascular events reported in treated patients, led to the non-approval of the drug in Europe.

**Fig. 1:**
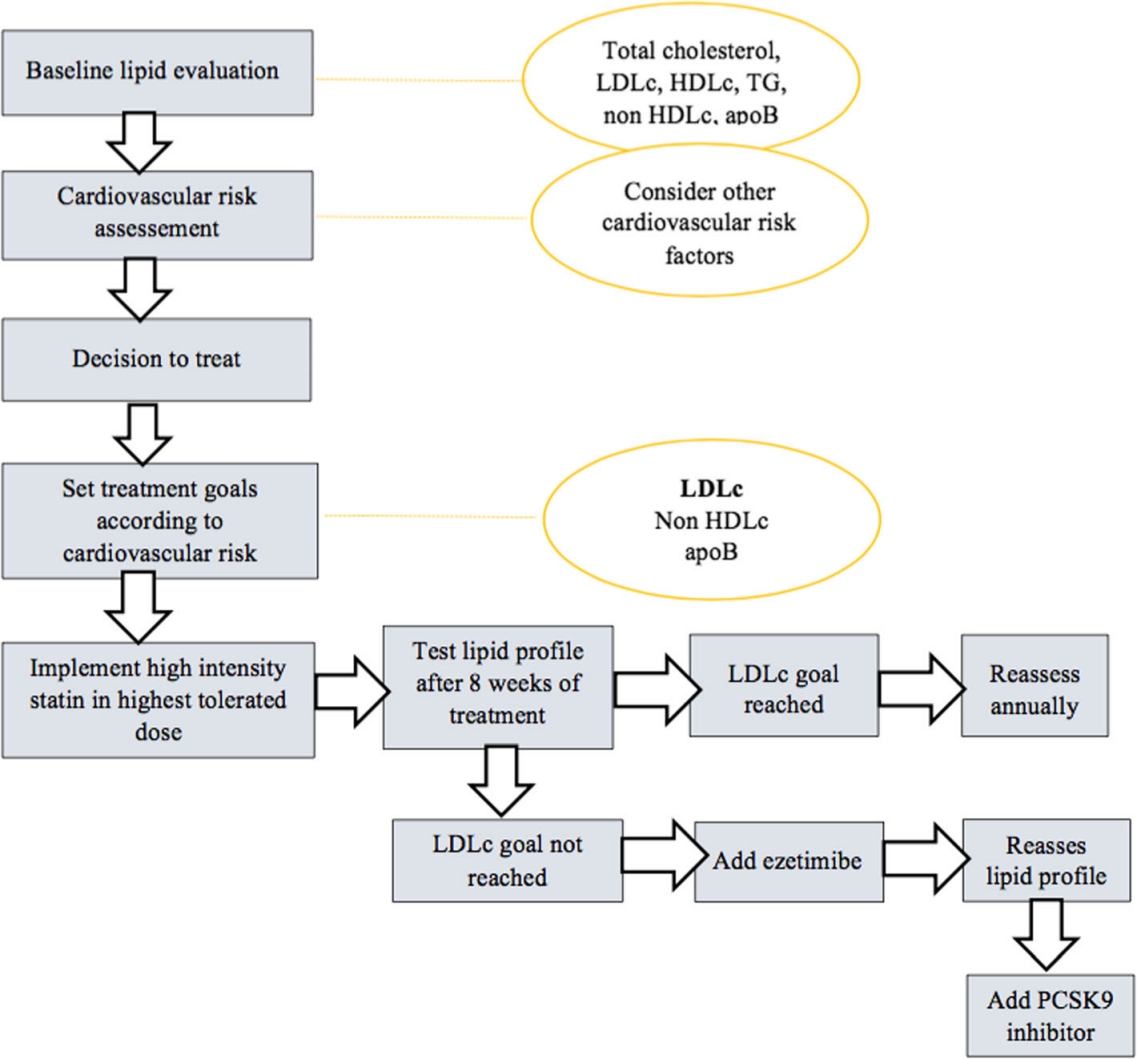
Recommended diagnostic and treatment algorithm of dyslipidemia

Fig. 1 presents a synthesis of the current recommended approach in patients with dyslipidemia. Table 1 summarizes the advantages and disadvantages of hypolipidemic drugs.

**Table 1:** Advantages and disadvantages of hypolipidemic drugs

Molecules	Advantages	Disadvantages
Statins	- Extensively studied - Reduction of LDLc, but also pleiotropic effects - Beneficial effect on cardiovascular morbidity and mortality	- Adverse effects such as myopathy, usually mild adverse effects on liver function Several important drug interactions
Cholesterol absorbtion inhibitors	- Added to statin therapy, reduces LDLc levels - Useful when statin therapy not possible	- Same adverse effects as statins, while not worsening them in combination with statins
Bile acid sequestrants	- Reduction of LDLc, when added to statins - May reduce glucose levels in hyperglycaemic patients	- Gastrointestinal adverse effects - May increase TG levels - Drug interactions
Nicotinic acid	- Raise of HDLc and ApoA1	- Benefits not entirely proven in studies
Fibrates	- Substantial decrease of TG, moderate decrease of LDLc and increase of HDLc	- Gastrointestinal adverse effects - Raise of creatinine and homocysteine levels
PCSK9 inhibitors	- Powerful effect on LDLc levels, in line with cardiovascular risk reduction	- Administration by subcutaneous injections - High costs - Limited indications
Mipomersen	- Reduction of LDLc in homozygous familial hypecholesterolemia	- Administration by subcutaneous injections - Liver toxicity - Not approved in Europe
Lomitapide	- Reduction of LDLc in homozygous familial hypecholesterolemia - Also approved in Europe	- Hepatic adverse effects

### Particular clinical settings

#### Dyslipidemia in patients with chronic kidney disease

Patients with chronic kidney disease (CKD), irrespective of the disease stage – terminal, dialysis, renal graft – have a very high cardiovascular risk. Dyslipidemia, frequent in this population, is an important risk factor. Moreover, dyslipidemia can worsen the renal function via lipid deposits in the kidneys, with subsequent glomerulosclerosis and loss of nephron function [35].

The lipid panel in these patients is a particular one, with high levels of TG and low levels of HDLc, while total cholesterol and LDLc are frequently normal. Although the severity of dyslipidemia increases as the renal disease progresses, the lipid profile is not different in dialysis patients compared to non-dialysis-dependent patients. There are significant differences in patients with nephrotic syndrome, whose lipid profile is much more atherogenic and is characterized by higher levels of total cholesterol and LDLc, TG and lipoprotein(a). The mechanism leading to this peculiarity is multifactorial and includes the stimulation of apoB synthesis, the reduction of lipoprotein metabolism and the reduction of clearance.

In patients with renal graft, the control of cardiovascular risk factors (including dyslipidemia) is crucial, as cardiovascular diseases are the main cause of death in this population. Pre-existent dyslipidemia can worsen after the initiation of immunosuppressive drugs. Cyclosporine reduces LDLc binding and uptake in the liver by inhibiting the LDLc receptor-mediated pathway; this leads to a rise in the level of total cholesterol and LDLc and to a drop in the level of HDLc, a dose-dependent alteration [36]. Corticoids can induce dyslipidemia by promoting the hepatic synthesis of lipids, especially VLDL synthesis, as well as by diminishing the hepatic uptake of LDLc. Tacrolimus and azathioprine seem to induce less important alterations of the lipid profile and can be considered for replacement of the more harmful cyclosporine.

Considering the high cardiovascular risk in patients with CKD, the treatment of dyslipidemia seems a logical approach. The use of statins as a first-line treatment has shown clear benefits. One meta-analysis of 38 studies including more than 37,000 patients with non-dialysis-dependent CKD showed a significant reduction of major cardiovascular events, all-cause mortality, cardiovascular death and myocardial infarctions in patients treated with statins versus controls. The treatment with statins did not influence the progression of the kidney disease [37]. The European guidelines recommend treatment with statins or statin/ezetimibe combinations in patients with CKD non-dialysis dependent, as these patients (stage 3–5 of CKD) have a high or a very high cardiovascular risk (class IA recommendation) [6].

Different statins have different renal clearances. Most of the statins are mainly metabolized by the liver, so no adjustment is necessary in the initial stages of CKD. However, at a creatinine clearance below 30 mL/min, some statins impose dose reductions. Atorvastatin and simvastatin do not require dose adjustments in patients with GFR < 30 mL/min, but this is not the case of pravastatin and rosuvastatin (maximal doses of 10 mg and 20 mg, respectively).

Patients who need dialysis have no indication for statin treatment if atherosclerotic cardiovascular disease is absent (class III indication) [6]. The lack of indication is based on a number of studies that analyzed the role of statins in hemodialysed patients whom did not benefit from statin treatment. The reduction in cholesterol and LDLc was similar to that of non-dialysis patients but, unlike the latter, there was no significant reduction of cardiovascular mortality, myocardial infarction or stroke [38, 39]. No severe adverse effects were reported either. Although surprising, these results can be explained by the presence of other non-lipid atherogenic factors as the main cause of the development/progression of atherosclerosis, which makes dyslipidemia treatment less beneficial than in other CKD stages.

Regarding new therapies, apparently PCSK9 inhibitors have a similar effect on lowering the LDLc level, apoB and non-HDLc in patients with CKD stage 3–5 (CrCl 30–59 mL/min/1.73 m^2^) and in patients with a normal renal function. The safety profile is also convenient, without further worsening of the renal function [40, 41]. However, a cardiovascular risk reduction is yet to be proven and patients with a CrCl < 30 mL/min/1.73 m^2^ were not recruited in studies. Data concerning these drugs are not yet conclusive for this category of patients and therefore the European guidelines do not include any recommendation.

### Autoimmune disorders

Autoimmune disorders are associated with a high risk of atherosclerotic cardiovascular risk, as a consequence of the interaction between traditional risk factors and chronic inflammation, which leads to early atherosclerosis. As a result, numerous studies have focused on the analysis and on the control of risk factors in this category of patients.

Although the levels of LDLc and total cholesterol are not always significantly elevated in these patients, the HDLc level is disproportionately lower than the level of total cholesterol, which leads to a very atherogenic profile. An exception is systemic lupus erythematosus (SLE), where the pattern of dyslipidemia is similar to the general population with a high cardiovascular risk – high level of LDLc, total cholesterol and apoB and low levels of HDLc. Besides, SLE has the highest cardiovascular risk of the autoimmune disorders, as patients with a disease duration of over 5 years have a myocardial infarction risk 52 times higher than the control group [42]. The alterations in the lipid profile seem to be more important in active disease, but significant changes can be seen also in patients with inactive disease, suggesting the presence of other etiological factors besides inflammation [43].

Currently, there is no recommendation for starting statin treatment based only on the presence of rheumatic autoimmune disease [6]. The use of lipid-lowering drugs in these patients has the same recommendations as in the general population. In the primary prevention, the same criteria and risk scores are used and the treatment goals are not different. Considering the high cardiovascular risk, some authors suggested lower treatment thresholds or labeling patients with active disease in a higher risk category (same as patients with diabetes, for example) [44, 45]. Adequate control of the main disease is essential, given the correlation between disease activity and cardiovascular disease. Moreover, immunosuppressive treatment – corticoids, disease-modifying anti-rheumatic drugs (DMARDs) and biological agents (anti-TNF-α) –, by suppressing inflammation, leads to the amelioration of the lipid panel, mainly by raising HDLc levels [46, 47].

### Elderly patients

Since life expectancy has increased, there is an increasing need for therapeutic strategies for the elderly population. Although improvements in the management of cardiovascular diseases have been made, there is still a high prevalence of cardiovascular risk factors in the elderly, with dyslipidemia making no exception.

Clinical studies in this population group (especially > 80–85 years) are limited, and most of the recommendations are supported by subgroup analyses in randomized, controlled trials. In the secondary prevention, the treatment of dyslipidemia with statins in patients over 65 years old has shown the same favorable results, similarly to younger patients [48, 49]. Therefore, in the secondary prevention of cardiovascular events in the older people with cardiovascular disease, the treatment of dyslipidemia follows the same indications as in younger patients (class IA recommendation) [6].

In the primary prevention, the European guidelines recommend statins for patients over 75 years with high cardiovascular risk (class IIB) [6]. However, this recommendation is controversial, as it is not based on the same solid evidence as in the secondary prevention. Meta-analyses of studies in the primary prevention has shown modest benefits. One of these, including 70,000 patients, has shown some benefits, especially regarding all-cause mortality and major coronary and cerebrovascular events in patients over 65 years old, without reaching statistical significance [50]. Another recent meta-analysis showed a reduction in the incidence of myocardial infarction with almost 40% and of stroke with almost 25%, without any influence on general survival [51].

In addition, drug-drug interactions must be considered. These are more important in older patients with multiple comorbidities, who are prescribed numerous medications and have modified pharmacodynamics and pharmacokinetics. The patients’ compliance tends to be lower because of the adverse effects of statins, but also the doctors` attitude is different, as they tend to have concerns in prescribing the adequate doses for older patients. The treatment must be initiated in small doses and carefully up-titrated until reaching the recommended doses to acquire the optimal level of LDLc.

Regarding the adverse effects, musculoskeletal problems (from muscle pain to myopathies) do not seem to be more frequent in older patients. An analysis of five studies concerning the efficiency and safety of intensive statin therapy in older patients with coronary disease showed only 13 reported cases of myopathy, rhabdomyolysis and creatine kinase levels >10x the normal limit [51]. Cognitive side effects and even aggravation of pre-existent dementia in older patients have been described, but further studies that analyzed the effect of statins on the cognitive status did not confirm a potential effect on dementia, Alzheimer’s disease or cognitive function [52].

Although the use of statins in the primary prevention does not seem to increase survival, their use in patients older than 65 years does not seem to be harmful. Polypharmacy and possible drug-drug interactions represent the main disadvantages in these patients, yet some treatment benefits still exist.

## Conclusions

Data derived from studies revolving around the pathophysiology of atherosclerosis and from clinical trials supports the causal role of LDLc in atherosclerotic diseases. Thus, reducing LDLc levels represents the key element in the prevention and treatment of cardiovascular diseases. Statins are already the standard treatment and ezetimibe is an important adjuvant for an additional lowering of cardiovascular risk. However, given the significant residual cardiovascular risk, even under optimal treatment with statins, as well as the important number of patients who do not tolerate statins, the search for further lipid-lowering therapies is ongoing. Although until now only PCSK9 inhibitors entered the European guidelines, multiple other molecules are evaluated in studies currently in progress, with promising results so far. The future management of dyslipidemia will probably include not only a larger number of therapeutic options, but also different treatment recommendations according to patients’ particularities and their risk factors.

## Data Availability

Not applicable.

## Abbreviations

ApoB: Apolipoprotein B
CrCl: Creatinine clearance
DMARDs: Disease-modifying anti-rheumatic drugs
EMA: European Medicines Agency
FDA: Food and Drug Administration
HDLc: High-density lipoprotein cholesterol
HMG CoA: β-Hydroxy β-methylglutaryl-CoA
LDLc: Low-density lipoprotein cholesterol
LDLR: Low-density lipoprotein cholesterol receptor
PCE: Pooled Cohort Equations
PCSK9: Pro-protein convertase subtilisin/kexin type 9
PPARα: Peroxisome proliferator-activated receptor alpha
SCORE: Systematic Coronary Risk Evaluation
SLE: Systemic lupus erythematosus
TNF-α: Tumour necrosis factor α
TG: Triglycerides
VLRL: Very low-density lipoproteins
